# Multiwell capillarity-based microfluidic device for the study of 3D tumour tissue-2D endothelium interactions and drug screening in co-culture models

**DOI:** 10.1038/s41598-017-12049-4

**Published:** 2017-09-20

**Authors:** María Virumbrales-Muñoz, José María Ayuso, Marta Olave, Rosa Monge, Diego de Miguel, Luis Martínez-Lostao, Séverine Le Gac, Manuel Doblare, Ignacio Ochoa, Luis J. Fernandez

**Affiliations:** 1 Department of Biomedical Engineering, Wisconsin Institutes for Medical Research, University of Wisconsin-Madison, 1111 Highland Avenue, Madison, 53705 Wisconsin United States; 2 Medical Engineering, Morgridge Institute for Research, 330 N Orchard Street, Madison, 53715 Wisconsin United States; 3 Group of Applied Mechanics and Bioengineering (AMB), Centro de Investigación Biomédica en Red. Bioingenieria, biomateriales y nanomedicina (CIBER-BBN), Mariano Esquillor Gómez, Zaragoza, 50018 Spain; 4 Aragon Institute of Engineering Research (I3A), University of Zaragoza, Mariano Esquillor Gómez, Zaragoza, 50009 Spain; 5 Aragon Institute of Biomedical Research, Instituto de Salud Carlos III, Mariano Esquillor Gómez, Zaragoza, 50009 Spain; 6 BEONCHIP S.L., Mariano Esquillor Gómez, Zaragoza, 50018 Spain; 7 Centre for Cell Death, Cancer and Inflammation (CCCI), UCL Cancer Institute, University College of London, Gower Street, London, WC1E 6BT UK; 8 Department of Biochemistry, Molecular and Cell Biology, University of Zaragoza, Calle de Pedro Cerbuna, 12, Zaragoza, 50009 Spain; 9 Aragon Institute of Biomedical Research (IIS Aragón), Instituto de Salud Carlos III, Avda. San Juan Bosco 13, Zaragoza, 50018 Spain; 10 Department of Microbiology, Preventive Medicine and Public Health, University of Zaragoza, Domingo Miral, Zaragoza, 50009 Spain; 11 Department of Immunology, University Clinical Hospital Lozano Blesa, Padre Arrupe, Zaragoza, 50009 Spain; 12 Institute of Nanoscience of Aragón (INA), Mariano Esquillor Gómez, Zaragoza, 50009 Spain; 13 Applied Microfluidics for BioEngineering Research, MESA+ Institute for Nanotechnology, MIRA Institute for Biomedical Research and Technical Medicine, University of Twente, Enschede, The Netherlands

## Abstract

The tumour microenvironment is very complex, and essential in tumour development and drug resistance. The endothelium is critical in the tumour microenvironment: it provides nutrients and oxygen to the tumour and is essential for systemic drug delivery. Therefore, we report a simple, user-friendly microfluidic device for co-culture of a 3D breast tumour model and a 2D endothelium model for cross-talk and drug delivery studies. First, we demonstrated the endothelium was functional, whereas the tumour model exhibited *in vivo* features, *e*.*g*., oxygen gradients and preferential proliferation of cells with better access to nutrients and oxygen. Next, we observed the endothelium structure lost its integrity in the co-culture. Following this, we evaluated two drug formulations of TRAIL (TNF-related apoptosis inducing ligand): soluble and anchored to a LUV (large unilamellar vesicle). Both diffused through the endothelium, LUV-TRAIL being more efficient in killing tumour cells, showing no effect on the integrity of endothelium. Overall, we have developed a simple capillary force-based microfluidic device for 2D and 3D cell co-cultures. Our device allows high-throughput approaches, patterning different cell types and generating gradients without specialised equipment. We anticipate this microfluidic device will facilitate drug screening in a relevant microenvironment thanks to its simple, effective and user-friendly operation.

## Introduction

Our understanding of cancer has undergone significant changes in the last years. We used to think of cancer as a clonally expanded cell population originated from a single cell^1^. However, it is now evident that tumours consist of highly complex systems, where the tumour microenvironment plays a fundamental role^2^. Spatial and temporal complexities (such as biochemical signals and gradients thereof), together with the variety of cell types and phenotype differences within the same cell type add up to compose this orchestrated environment in tumour tissues, which is known as the tumour microenvironment (TME)^2–5^. It has been previously reported that this high complexity and heterogeneity play a significant role in drug resistance. Moreover, tumour cells also interact with the stroma, conditioning the initiation of the disease, development, and resistance to therapy^6^.

Furthermore, the endothelium plays a significant role in cancer development and drug delivery. The endothelium regulates nutrient diffusion and responds to hypoxic signals produced by tumour cells to promote new blood vessel formation via angiogenesis. Additionally, blood vessel permeability is essential for the delivery of drugs to cancer cells, which, in turn, determines the overall effect of the drug on the patient^7,8^. Of particular interest is the Enhanced Permeability and Retention (EPR) effect, which is caused by the generation of a porous architecture in the blood vessels surrounding the solid tumours, leading to an increased vascular permeability. The EPR effect results in more efficient oxygen and nutrient supply to the tumour sites than from regular vessels^8^. Likewise, nanomedicine-based treatments take advantage of this EPR effect for the local accumulation of nano-vehicled agents, which are meant for diagnostic or therapeutic purposes^8^. Currently, the delivery and efficiency of treatments against cancer disease are still mostly tested in classical *in vitro* 2D models^9–11^, which usually lack an extracellular matrix, do not include different cell types arranged in a structured manner and lack biochemical gradients (*e*.*g*., of oxygen and nutrients). Consequently, 2D models are unable to faithfully reproduce the tumour-endothelium interactions, where crosstalk depends on a 3D environment, as well as on direct cell-cell interactions in a 3D manner. Of particular interest for the generation of sophisticated models are the so-called organ-on-a-chip platforms, which combine different cell types and microfabricated structures and allow mimicking the architecture of a whole organ and/or tissues^12^. Such microfluidic technology shows great potential in the field of cancer research to faithfully mimic the TME^13^. To date, significant efforts in the field of microfluidics have focused in reproducing the tumour microenvironment, and, in particular, studying the interactions of cancer cells and the endothelium^14–17^. For instance, the Kamm group has established co-culture of breast cancer cells and endothelial cells to study cellular extravasation and vascular permeability^18^, as well as tissue-specific metastasis^15,19^. Additionally, they successfully characterised molecular mechanisms of metastasis in breast cancer^20–22^, and macrophage extravasation. Also worth mentioning are the efforts in establishing distinctively patterned co-cultures made by the Beebe group for drug screening and biosensing applications^23,24^. Interested readers in microfluidic cell culture approaches in cancer are referred to excellent reviews by Sung *et al*., Portillo-Lara *et al*. and Regier *et al*.^25–27^.

From these different examples, we can infer that despite all these efforts, microfluidic technology remains restricted to engineering laboratories and has mainly focused on proof-of-concept applications. The fact that microfluidics is rarely used in traditional cell biology laboratories can be explained by the high difficulty of operation and low- or medium-throughput of most microdevices^28,29^. Therefore, to bridge the gap between microfluidics and basic and translational research laboratories, a new generation of microdevices is needed, which balances the simplicity of operation, reproducibility, biological relevance and usefulness in the described contexts. Additionally, integration of endothelial layers in microfluidic models has also proven difficult. This integration requires a continuous interface to provide support and allow endothelial cells to attach correctly. Furthermore, a common method to assess endothelium integrity *in vitro* relies on TEER (TransEpithelial Electrical Resistance) measurements^30,31^. Nonetheless, this approach presents a few drawbacks, which limit its applicability. Namely, it requires the integration of an electrical setup within the biological setup; it requires specific electrodes to be built-in or added externally to the microfluidic system; and, more importantly, the lack of stability for these measurements is a well-known problem, which yet, remains to be overcome^32^. Optimised optical inspection and easy access to the endothelium would provide not only an advantageous alternative but also additional information on cell morphology and tight junctions in the endothelium using immunofluorescent staining.

**Figure 1:**
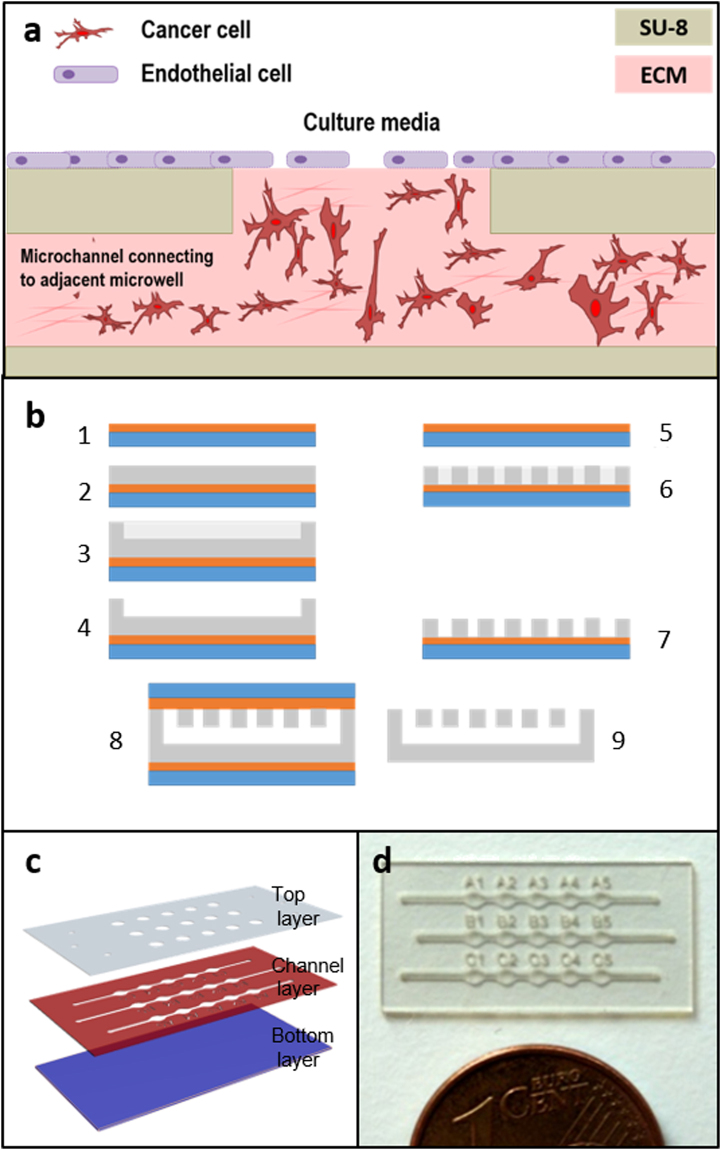
Experimental configuration and microdevice fabrication: (**a**) Scheme of the 3D tumor-2D endothelium co-culture model recreated in the present study within the device; (**b**) Chip fabrication process flow: (1) Kapton film bonding to a pyrex wafer, (2) deposition of a 90-µm thick SU-8 layer, UV-exposure and polymerization of the microchannel and microwell structures, (3) spinning of a 90- µm thick SU-8 layer and patterning of the microchannel and microwell structures, (4) development of the two SU-8 layers, (5) Kapton film bonding to a pyrex wafer, (6) spinning of a 90-µm thick SU-8 layer and patterning by photolithography, (7) cover development, (8) SU-8 to SU-8 thermal bonding, (9) SU-8 device release. (**c**) Schematic representation of an open device, showing the different SU-8 layers: the bottom layer (blue) the channel layer (red), including the channels and microwells and a top layer (grey) with microwell structures to yield open microwells and closed microchannels; (**d**) Photography of a final microdevice consisting of 3 arrays of 5 microwells each.

In this context, we present here a simple and self-filling SU-8-based microdevice design, which exploits capillary forces, to study endothelium-tumour interactions. The proposed design consists of several linear arrays of microwells (Fig. 1c), in which 3D tumour models are created by embedding tumour cells in a 3D collagen matrix and, on top of which confluent HUVEC monolayers are prepared as 2D mimics of the endothelial barrier. Although similar approaches have already been reported^33^, our device allows filling an array of microwells in only one single pipetting step and a few seconds, fulfilling thereby the key requirements of simplicity of operation and user-friendliness. Additionally, the design of the microdevice has been optimised for optical examination of the endothelium to evaluate its integrity. This approach can replace TEER measurements for an easier and more comprehensive approach to endothelium integrity. Here, we first demonstrated co-culture of breast tumour cells (MDA-MB-231) seeded in 3D with an endothelium (HUVEC) and thoroughly characterised these models (Fig. 1a). Next, we applied our model to study the cytotoxic effects of drugs and their penetration in the 3D tumour environment. To that end, the anti-tumour agent TNF-related apoptosis-inducing ligand (TRAIL) was evaluated. TRAIL is a protein secreted by immune cells, and which can induce apoptosis in malfunctioning cells^34^. *In vivo*, TRAIL is secreted as anchored to liposomes, and this clustered form has been found to have a higher potential for inducing apoptosis^35^. Here, two different drug formulations were therefore tested; a soluble version (sTRAIL) and an “improved” formulation based on liposomes decorated with TRAIL (LUV-TRAIL). Altogether, we have developed a simple and highly versatile microfluidic device design with a physiologically relevant tumour-endothelium co-culture model, which allows testing the penetration and efficiency of drugs on tumour tissues.

## Results

### Hydrophilic features and liquid handling within the microdevice

**Figure 2:**
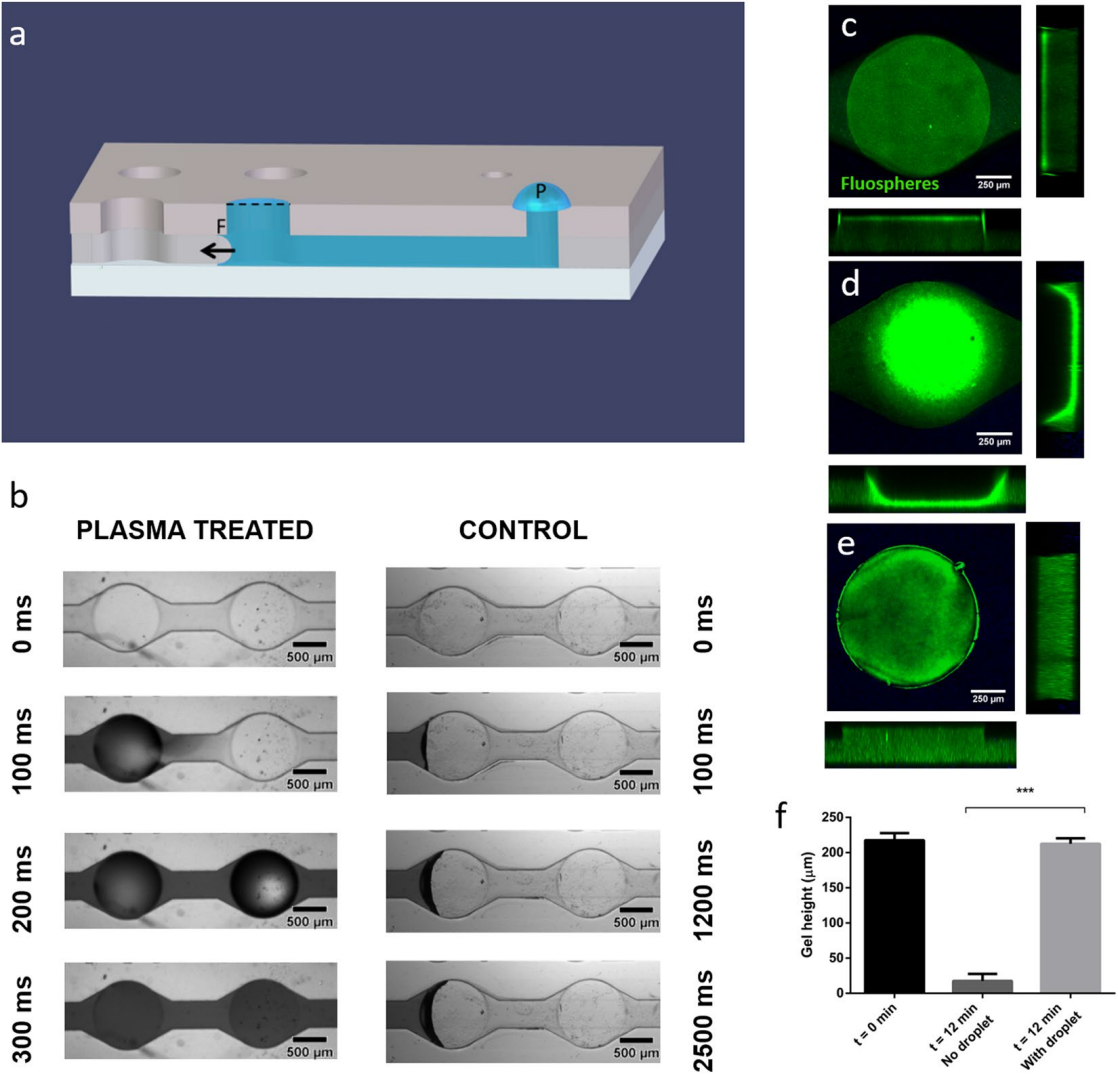
Air-hydrogel interface analysis and optimization in the microdevice. (**a**) Schematic representation of the device, showing capillary forces exerted by the geometry, and capillary valves integrated into the design (dotted line). The scheme includes the hydrogel droplet that was placed on the top of the main inlet, to compensate for evaporation issues of the solution in the hydrogel and to ensure the formation of a flat hydrogel-air interface. (**b**) Influence of the plasma treatment on the microdevice spontaneous filling. Left- Bright-field 20x images showing the filling dynamics of a microdevice after plasma treatment at 0, 100, 200 and 300 ms, respectively; Right- Bright-field 20x images showing the filling dynamics of a microdevice without any plasma treatment at 0, 100, 1200 and 2500 ms, respectively. (**c**-**f**) Characterization of the hydrogel-air interface in the microdevice using confocal microscopy. 100x images flanked by z-stack orthogonal views of the gel interface at 0 min after hydrogel introduction (**c**) and 12 min after introduction of the hydrogel, with (**e**) or without addition of a droplet of water (**d**). Water evaporation caused a dramatic contraction of the hydrogel (**d**) and FluoSpheres® accumulation at the interface, which formed a deep meniscus in the well. (**e**) Hydrogel polymerized with an additional hydrogel droplet placed on the main inlet during 12 min. No meniscus was observed and the hydrogel-air interface was flat as desired. (**f**) Quantification of the hydrogel height in the microwells just after introduction of the hydrogel (t = 0 min) and 12 min later, after gel polymerization, when no droplet was added on the inlet (t = 12 min. No droplet) and when a droplet was added on the inlet (t = 12 min with droplet). (***) p < 0.001 as calculated through Kruskal-Wallis after disproving data normality. The graph shows Average ± SEM – N = 10.

SU-8 was selected as a building material due to its previously reported biocompatibility in cell culture applications^36,37^, as well as some additional intrinsic advantages over native PDMS (Polydimethylsiloxane) for the fabrication of cell culture systems. These advantages include broader chamber design possibilities^38^, convenient mechanical properties^39^, monolithic integration with a variety of sensors and, importantly in our case, low gas permeability^40^, which facilitates the creation of an oxygen gradient without any need for external systems. SU-8, provides a high degree of control over the fabricated structures and capillary valves, as included here. Once the microdevices were fabricated (Fig. 1), they were treated with O_2_ plasma to promote spontaneous filling of the devices. Following this treatment, the contact angle was reduced from 82.6 ± 1.6° to 21.1 ± 2.3° (N = 10, Supplementary Fig. S1), and spontaneous hydrogel introduction was observed in the microwell arrays (Fig. 2b, left panels). This behaviour was also expected according to the microdevice design. Besides, spontaneous microdevice filling occurred in a few seconds, ensuring thereby that the hydrogel filled the microdevice completely before collagen polymerization would occur. Interestingly, in the absence of plasma treatment, capillary forces were not strong enough to fill the microdevice completely, and hydrogel injection was extremely slow. Consequently, the hydrogel remained pinned in the microchannels connecting the microwells without filling the entire microdevice (Fig. 2b, right panels).

### Air-hydrogel interface analysis and optimisation in the microdevice

Next, we characterised the air-hydrogel interface in the microdevice after collagen polymerization to ensure that a flat interface was created, which is essential for cell imaging. To that end, the microdevice was filled with collagen supplemented with FluoSpheres® and the interface was characterised both immediately after filling the microdevice and after collagen polymerization.

The interface was flat immediately after collagen perfusion (Fig. 2c), showing a homogeneous distribution of the FluoSpheres® in the hydrogel. However, after polymerization a 200 μm-deep meniscus appeared, causing the Fluosphere® microbeads to cluster in the centre at the interface (Fig. 2d). We assumed this meniscus was created due to water evaporation during the polymerization process at 37 °C within the incubator. Therefore, to avoid the formation of such a meniscus, we placed a hydrogel droplet at the device inlet (Fig. 2e) to counteract evaporation. As presented in Fig. 2a, the pressure exerted by the droplet was enough to maintain a flat air-liquid interface during the whole hydrogel polymerization process, by compensating any evaporation of the solution.

### Microdevice biocompatibility and characterization of the 3D cell culture microenvironment

**Figure 3:**
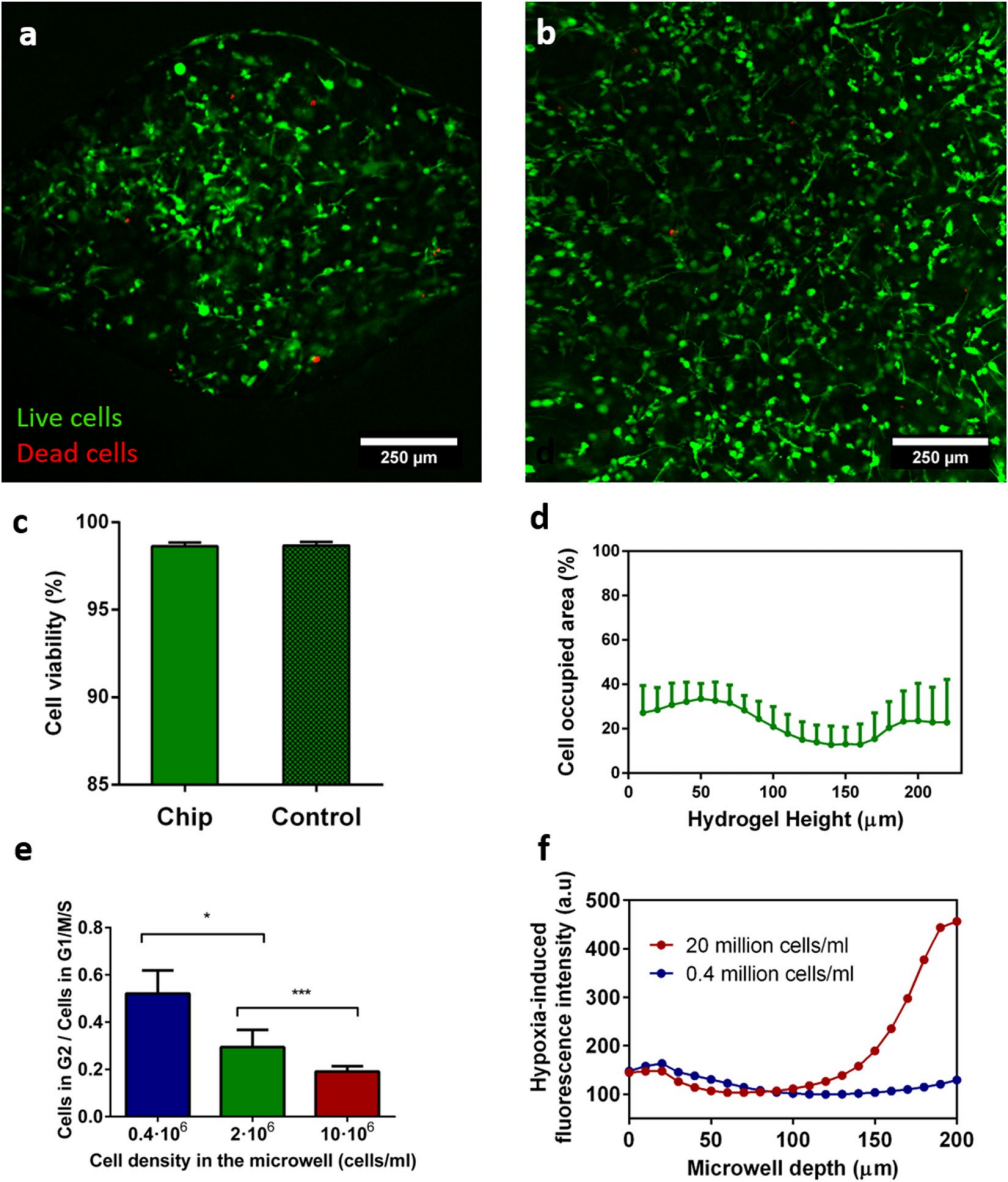
Viability of the MDA-MB231 cells grown in the microdevice in a 3D hydrogel matrix. Viable cells were stained with Calcein AM (green), whereas dead ones were labelled with propidium iodide (red). (**a**) 100x confocal image showing the cell viability in one microwell in the device. (**b**) 100x confocal image of the same cells grown in 2D on a Petri dish (control). (**c**) Quantification of the MDA-MB-231 cell viability within the microdevice and in Petri dishes. Welch’s t-test was performed for comparison (two-tailed distribution, α = 0.05) and non-significant statistical differences were found (N = 3). (**d**) Quantification of the cell occupied area as a function of the hydrogel depth. 0 µm refers to the top of the microwell (N = 10). (**e**) Proliferation index of cells cultured in the hydrogel in the microdevice, calculated by normalizing the number of cells in G2/S/M (as characterized by Premo FUCCI transfection *in situ*) with the total number of seeded cells. Data analysed by means of Kruskal-Wallis’ Test (N = 3). (***) p-value < 0.0001. (**f**) Characterization of the oxygen level in the device for different cell densities (0.4·10^6^, 2·10^6^ and 10^7^ cells/ml) using Hypoxia-IT®. All graphs show average ± SEM.

As a next step, cell viability was examined in 3D culture in the collagen to confirm the device biocompatibility. Therefore, we seeded MDA-MB-231 breast tumour cells at a final density of 5 × 10^6^ cells/ml in a 1.2 mg/ml collagen hydrogel. After three days in culture, cell viability was assessed with calcein AM (green) and propidium iodide (red). Through this method, live cells were stained in green, whereas dead cells were stained in red. Cell viability inside the microdevice was similar to that observed for control hydrogels also loaded with the same number of cells but cultured on a Petri dish (Fig. 3a–c), and in both cases, a cell viability higher than 98% was observed. Additionally, we evaluated the three-dimensional cell distribution along the microwell height. Typically, cells would occupy between 15 and 30% of the surface area of the microwell in each 10 µm-thick layer imaged by confocal microscopy, and no significant difference was observed throughout the whole well height (Fig. 3d). Altogether, there was no significant accumulation of cells at a specific height in the collagen in the microwells, and no sedimentation of the cells at the bottom of the microwells.

Furthermore, we characterised the tumour cell behaviour and function within the microwells. Firstly, we investigated cell proliferation for different cell seeding densities (0.4 × 10^6^, 2 × 10^6^ and 10^7^ cells/ml) using Premo® FUCCI Cell Cycle Indicator, which stains G0/G1 phase cells in red and S, G2 and M phase cells in green. MDA-MB-231 tumour cells embedded in the collagen gel were transfected with this proliferation marker within each independent array of microwells in a single microdevice, and the proliferation index of cells was determined as the number of cells in G2/M/S divided by the total number of seeded cells. Results revealed that the proliferation index significantly diminished for higher cell concentrations (Fig. 3e). Specifically, a 5-fold cell concentration increase (from 0.4 × 10^6^ to 2 × 10^6^ cells/ml) was accompanied by a reduction in proliferation to 53.3%, and a 4-fold increase in concentration by a 28.7% decrease in proliferation (Supplementary Fig. S4).

Proliferation differences can be accounted for variations in nutrient and oxygen availability. To test this hypothesis –*i*.*e*., if there was a significant change in oxygen concentration depending on the cell concentration, we checked for the possible depletion of oxygen within the microwells. For this experiment, we compared microwells prepared with either high or low cell concentrations in collagen hydrogels. The graph presenting these results in Fig. 3f indicates significantly different oxygen concentration profiles for the two cell densities tested here (0.4 × 10^6^ and 10^7^ cells/ml). For a low cell concentration (0.4 × 10^6^ cells/ml), a low and constant fluorescence level was found throughout the entire height of the microwell after 24 h, indicating a normal oxygen level. In contrast, a gradient in oxygen concentration was observed for the high cell concentration (10^7^ cells/ml) with marked oxygen depletion at the bottom of the well after 24 h of culture under the same conditions (Fig. 3f). Altogether, the culture of cells in the hydrogel in the microwells beyond a certain cell concentration threshold resulted in the appearance of deprived oxygen conditions, which could explain the marked decrease we record regarding cell proliferation, as initially hypothesised. Furthermore, with our model, we were able to mimic the typical architecture found in tumour tissues *in vivo*, which typically contain a hypoxic non-proliferating core and a proliferative periphery with direct access to oxygen^41^. This structure is particularly relevant to assess the efficiency of drugs, since some drugs are more effective on proliferating cells and not active under hypoxic conditions, while other drugs are specifically activated in an oxygen-poor environment^42^.

### Characterisation of the endothelial barrier model

The second main component of the co-culture is the endothelium layer on top of the 3D culture in each microwell. The endothelium was created by seeding HUVEC cells (firstly phenotyped, data available in Supplementary Fig. S2) at a density of 250,000 cells/cm^2^ on microwells in a microdevice previously filled with 1.2 mg/ml type I collagen, and letting them attach for 24 h, to create a confluent monolayer to mimic an endothelium. The endothelium integrity was first characterised through diffusion assays with two fluorescent dextrans (40 kDa and 70 kDa), as previously described in the literature^20^ (Supplementary Fig. S5). Diffusion rates were 1.91 ± 0.88 ·10^−5^ cm/s and 1.70 ± 0.81·10^−6^ cm/s, respectively (Supplementary Figs S5a–c & e–g), consistent with data previously reported in the literature. The diffusion profiles show a delayed penetration of the dextran particles through the monolayer, compared to control hydrogels (Supplementary Fig. S5d). These results indicate that the endothelial monolayer hinders the diffusion of molecules, as reported in the literature^20^. Furthermore, results do not significantly vary between 24 and 48 hours of seeding.

The endothelium integrity was further characterised by immunostaining for F-Actin, VE-cadherin, and Nuclei. These markers allowed us not only to assess the percentage of the microwell surface area that was covered with cells (F-actin) but also to demonstrate the existence of cell-cell adhesions (VE-cadherin), confirming the endothelium is not leaky.

**Figure 4:**
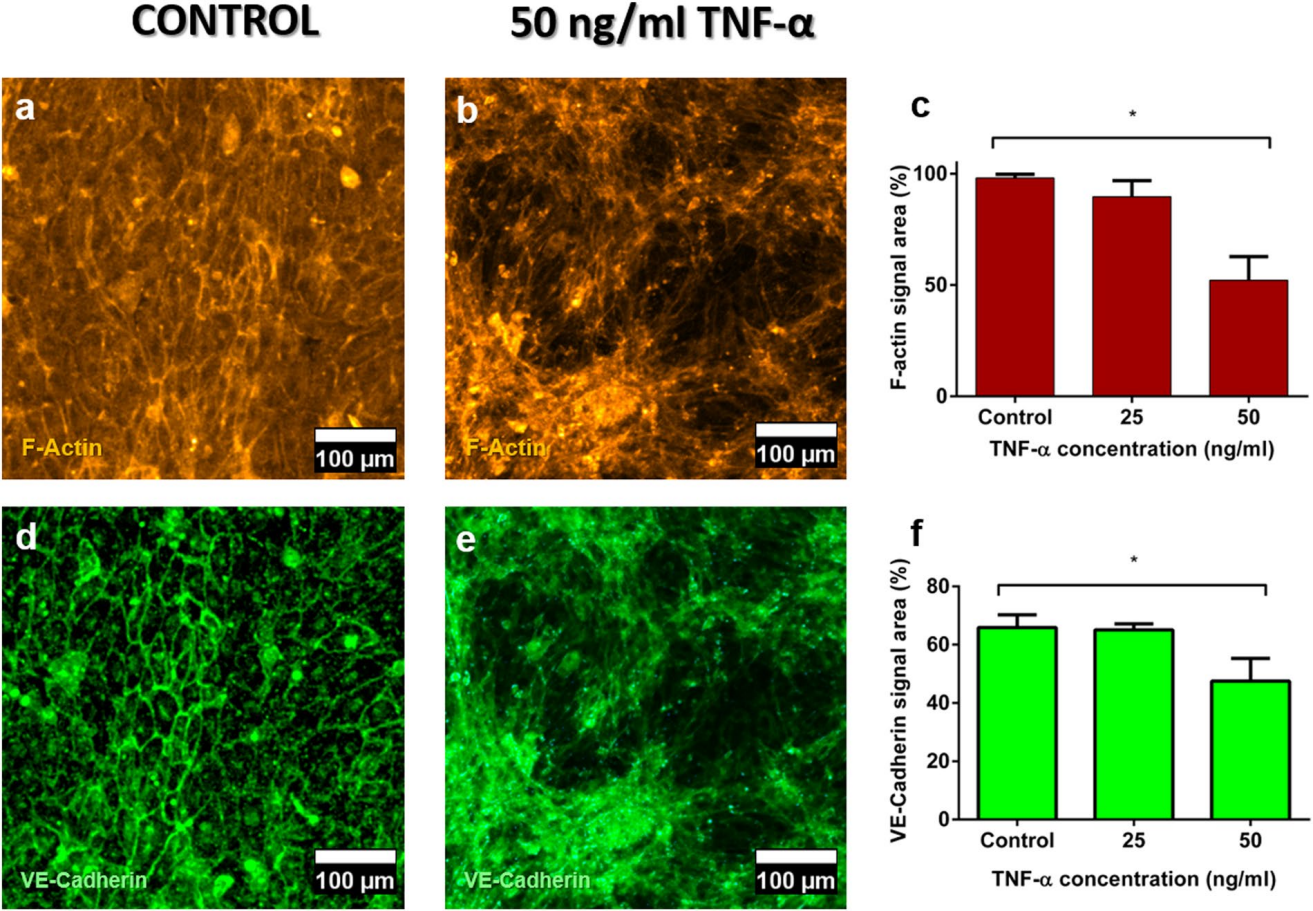
Characterization of the endothelium generated on the top of the hydrogel matrix in the microdevice. Endothelial cells (HUVECs) were labelled with Phalloidin-TRITC (yellow) for actin staining and VE-Cadherin (green) for endothelial cell junction staining. (**a** and **d**) Control endothelium; (**b** and **e**) Endothelium treated with 50 ng/ml TNF-α for 24 h (**c**) Quantification of the endothelium integrity in terms of Phalloidin signal area for all samples (not treated, and treated with 25 and 50 ng/ml TNF-α). Samples were compared with the control. (*) p-value: 0.02. (**f**) Quantification of endothelium integrity in terms of VE-cadherin signal area for all samples (not treated, and treated with 25 and 50 ng/ml TNF-α). Samples were compared with the control. (**) p-value for 50 ng/ml TNF-α = 0.005, as calculated by Kruskal-Wallis’ Test. Graphs show average ± SEM (N = 6) and magnification is 200x for all images.

As presented in Fig. 4, the hydrogel surface in the microwells was entirely covered by the HUVEC cells after 24 h, which had established cell-cell contacts across the complete area of the microwell (Fig. 4a,b, whole microwell images in Supplementary Image S6). Besides, no apparent gap was visible between the cells, indicating the formation of a tight endothelium after 24 h of culture. The endothelium integrity was further quantified through cell area measurements, by comparing the surface area occupied by cells (derived from the F-actin or VE-cadherin fluorescence signal) with the total surface area of a microwell (Fig. 4c,f, respectively). These measurements indicate that a tight endothelium was successfully created on top of each microwell.

We next evaluated the functionality of the endothelium model by treating it with pro-inflammatory factors such as TNF-α. TNF-α has been reported to increase endothelium permeability at concentrations of 25 and 50 ng/ml^43^. As expected, after 24 h exposure to TNF-α, we observed the creation of gaps in the HUVEC monolayer (Fig. 4b,e). Additionally, a quantitative decrease was observed in the area occupied by cells (Fig. 4c,f), as assessed for both VE-cadherin and F-actin staining, and this decrease was found statistically significant for a TNF-α concentration of 50 ng/ml. This assay confirms that the 2D endothelium model is entirely functional and responsive to inflammation, as previously described in the literature^20^.

### Co-culture model with a 2D endothelium and MDA-MB-231 breast tumour cells cultured in 3D in a collagen matrix

After the establishment and characterization of both the 3D tumour cell culture and the 2D endothelium, both elements were combined in the same microdevice.

**Figure 5:**
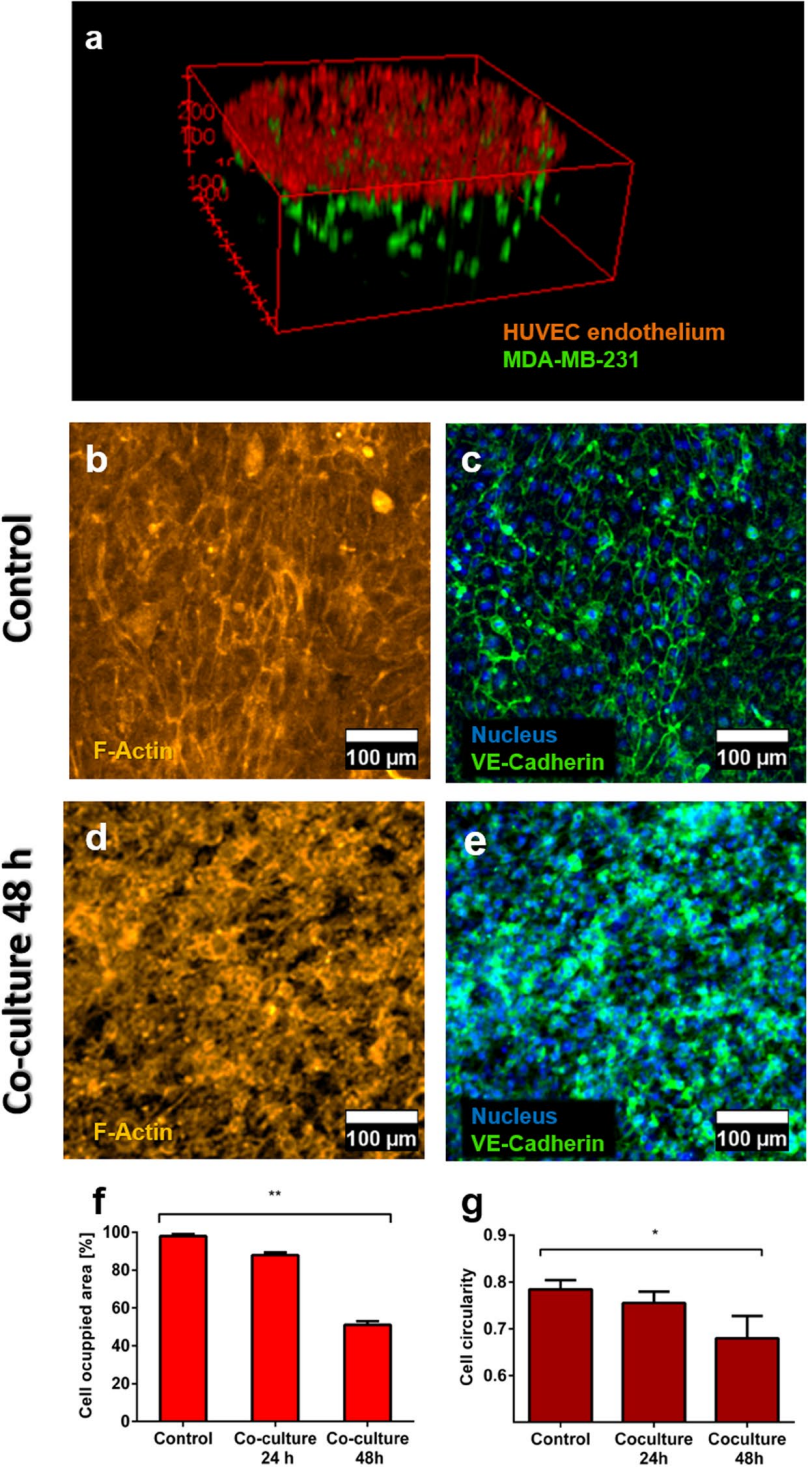
Co-culture of MDA-MB231 tumour cells with HUVECs cells. (**a**) 3D reconstruction of the 2D-3D co-culture model within the microdevice after 24 h of seeding, tumour cells being grown in the 3D hydrogel matrix and HUVECs as a monolayer on top of the hydrogel in the microwells. (**b**–**g**) Assessment of the integrity of the endothelium monolayer in the co-culture system, compared to control conditions (endothelium mono-culture). b- Actin staining of a control HUVEC endothelium (mono-culture). (**c**) Detail of a control HUVEC endothelium (mono-culture) stained with VE-Cadherin and NucBlue®. (**d**) Actin staining of a HUVEC endothelium in co-culture with MDA-MB-231 tumour cells, 48 h after cell seeding. (**e**) Detail of a HUVEC endothelium in co-culture with MDA-MB-231 tumour cells, 48 h after seeding, stained with VE-Cadherin and NucBlue®. (**f**) Assessment of the integrity of the HUVEC endothelium for the co-culture after 24 and 48 h compared to control conditions (mono-culture of a HUVEC monolayer) quantified as F-actin signal area. (n = 5, p < 0.02 as calculated with Kruskal-Wallis’ Test) (**g**) Assessment of the HUVEC cell circularity for control (mono-culture) and co-culture conditions after 24 and 48 h. Data was normally distributed and was evaluated by means of one-way ANOVA (n = 20). Graphs show average ± SEM and magnification is 200x for all images.

First, the respective HUVEC and MDA-MB-231 cell distributions were investigated by imaging and after cell membrane staining with two different Vybrant® cell trackers: MDA-MB-231 cells were labelled in green, and HUVEC cells in red (Fig. 5a). As observed in separate mono-culture conditions, a flat endothelium was created on the top of each microwell, with a homogeneous three-dimensional distribution of the tumour cancer cells in the collagen matrix under the endothelium layer. Up to 24 h after cell seeding, the endothelium was found to be integer and tight (Supplementary Figs S7 & S8), but as time passed, changes in the endothelium appearance and integrity were detected. Specifically, after 48 h of co-culture after, the shape of the endothelial cells evolved compared to control conditions (*i*.*e*., in the absence of tumour cells in the collagen), moving from a polyhedral shape towards a spindled one (Fig. 5e,f). The HUVEC cell circularity was further quantified in control conditions (mono-culture, 48 h) and after 24 h and 48 h of co-culture with the 3D tumour model (Fig. 5g), revealing a 4% decrease in cell circularity between the control and co-culture after 24 h (from 0.78 to 0.76). Moreover, the cell circularity decreased by 15% at 48 h of co-culture compared to the control (from 0.78 to 0.68). Next, the cell-cell connections in the endothelium disappeared, as indicated by a loss in the characteristic VE-cadherin distribution for blood vessels. The integrity of the endothelium was quantified as before, revealing a significant decrease by 52% compared to control conditions. Importantly, this decrease in F-actin signal is very similar to that observed for a monoculture endothelium treated with 50 ng/ml of TNF-α for 24 h (Fig. 4c). Finally, to distinguish HUVEC cells from MDA-MB-231 cells, the co-culture experiment was performed with HUVEC cells labelled with a cell tracker (CMFDA) and unlabeled cancer cells. The area occupied by HUVEC diminished over the course of 48 h, which confirms the previous results, indicating that cancer cells disrupted the integrity of the endothelial layer (Fig. S8). These results altogether demonstrate that co-culture of an endothelium with MDA-MB-231 breast tumour cells led to the creation of a leaky vasculature, as observed *in vivo* in tumour-associated vessels, which should also present an EPR effect^8,9,44^.

### Drug screening in the tumour-endothelium model

Since the proposed tumour-endothelium co-culture model exhibited this key characteristic of leaky endothelium, we decided to apply it for drug penetration assays and evaluating the EPR effect, which is particularly interesting for nanomedicines. For this drug assay, we chose the death ligand TRAIL (TNF-related apoptosis-inducing ligand), which was tested in its soluble form (60 kDa) and as a conjugate with a large unilamellar vesicle (LUV)^45^. Both forms were tested at a concentration of 0.33 ng/ml for 24 h in our co-culture model to evaluate their efficiency. As for the control, PBS (drug solvent) was added to the culture medium with the same amount as in the drug assay, to account for the dilution of the media.

**Figure 6:**
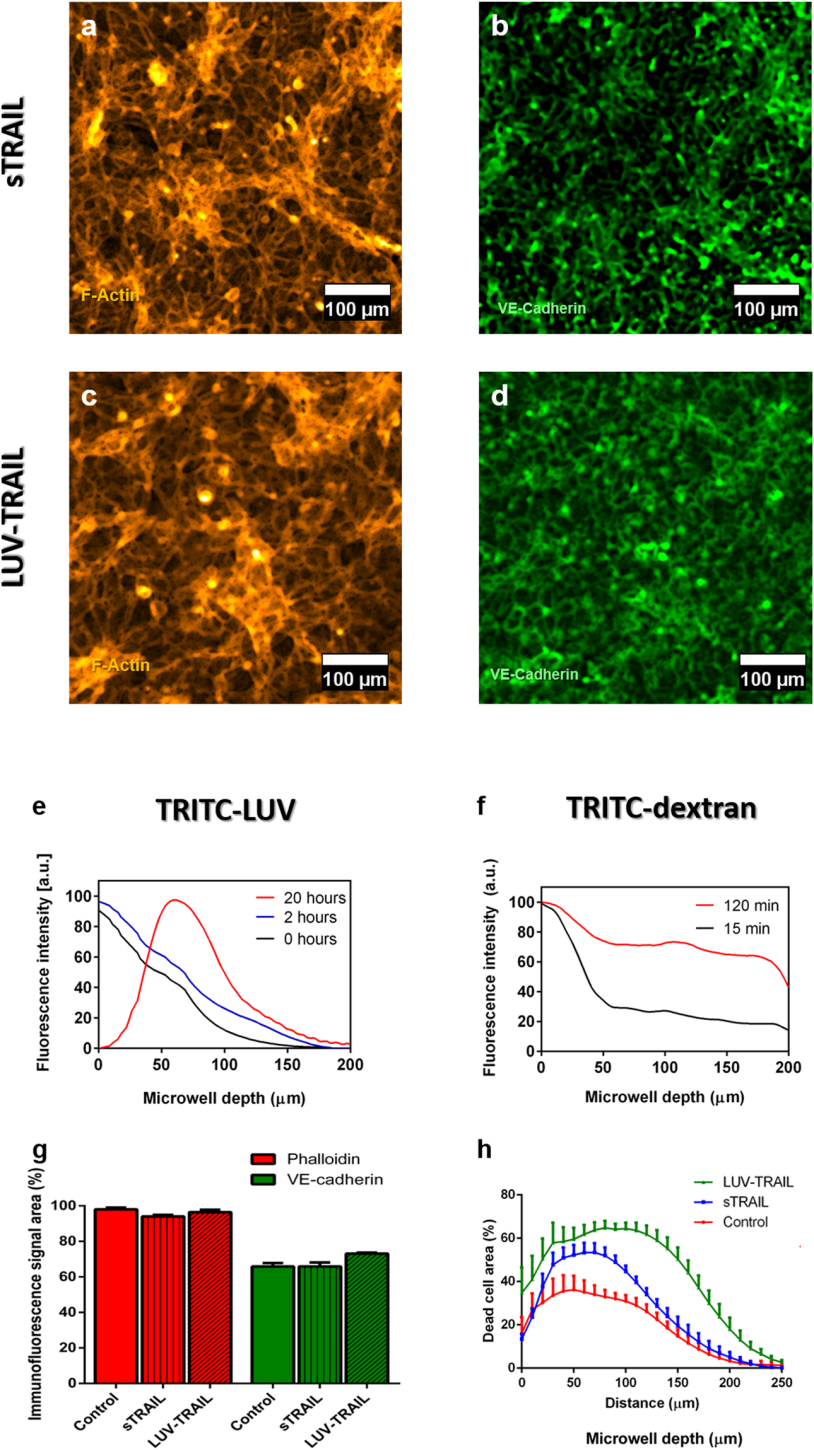
Drug testing assay using TRAIL in both its soluble form and anchored to a LUV in the co-culture established models. Endothelium immunofluorescence after 24 h of exposure to 0.33 ng/ml sTRAIL (**a**,**b**) and 0.33 ng/ml LUV-TRAIL (**c**,**d**) for Phalloidin-TRITC (yellow) and VE-Cadherin (green), respectively. (**e**,**f**) Diffusion/penetration assays using TRITC-labelled LUV (4 mg/ml) and TRITC-Dextran (70 kDa, 30 µM) used as models for the LUV-TRAIL and s-TRAIL, respectively, across an endothelium seeded on a collagen gel. (**g**) Quantification of the endothelium integrity using the Phalloidin (red) and the VE-cadherin (green) signal area for control conditions (no TRAIL exposure) and samples treated with both sTRAIL and LUV-TRAIL (p-value > 0.99 as calculated through Kruskal-Wallis’ Test). (**h**) Quantification of MDA-MB-231 tumour cell death in 3D along the device microwell depth in control conditions, and with sTRAIL and LUV-TRAIL treatment in the established microfluidic co-culture model. Here, 5 million MDA-MB-231 cells/ml were seeded in the hydrogel matrix. Graphs show average ± SEM. N = 7.

As a first step, the toxicity of both drug formulations was assessed on the endothelium alone. The drug effect on the endothelium was quantified as previously described in terms of changes in the endothelium integrity. No significant decrease in the cell occupied area was observed after treatment with both sTRAIL and LUV-TRAIL (Fig. 6a–d) using F-actin and VE-cadherin staining, compared to control monolayers (Fig. 6g). Nonetheless, a noticeable change in the fluorescence signal pattern was found, particularly in the case of F-actin expression (Fig. 6a,c) (whole microwell images available in Supplementary Image S9). Notably, F-Actin and VE-cadherin local accumulation and alignment in some regions of the microwell were observed, which is significantly different from the patterns obtained in control microwells. This observation is consistent with angiogenic and mitogenic events, as previously described in endothelial cells after TRAIL treatment^46^.

Before testing both drug formulations on the co-culture models, we assessed their penetration in the microwells using fluorescent surrogates such as dextran nanoparticles with a comparable size to sTRAIL (70 kDa) and a rhodamine-conjugated LUV as a model for LUV-TRAIL. As expected, the penetration rate was much lower in the case of the LUV, after both 15 min and 2 h, which is to be accounted for by the larger size of the LUV-based formulation. Nonetheless, both formulations could entirely penetrate across a healthy endothelium after 2 h (Fig. 6e,f), despite their size difference. Interestingly, the LUV vesicles seemed to aggregate and slowly sediment together after 2 h, since at 20 h they did not reach the bottom of the microwell. In contrast, the 70 kDa fluorescent dextran reached the bottom of the microwell within 2 h.

Finally, the two drugs were tested on the established co-culture model combining a HUVEC endothelium and a 3D tumour cell culture. Cell viability was evaluated as before in the collagen matrix using Calcein AM and PI, and quantified as the percentage of the microwell occupied by dead cells at each focal plane.

In absence of treatment (control conditions), moderate mortality was found. This result is consistent with the presence of a necrotic core, which would result from the presence of oxygen and nutrient gradients experienced by cells within the microdevice. Interestingly, after treatment of the co-culture with TRAIL for 24 h at 0.33 ng/ml, viability staining revealed that both drugs induced cell death throughout the entire microwell, with however a decay in cytotoxicity when going deeper in the microwell (Fig. 6h).

The slope at the beginning of the curve in Fig. 6h for both sTRAIL and LUV-TRAIL is similar to that observed in the control. As for the area under the curve, the control presents the smallest area under the curve of all conditions. Furthermore, the area under the curve for sTRAIL is 37% higher than the control (4908 ± 99.63 for the control and 6728 ± 83.59 for sTRAIL). As for LUV-TRAIL, the shape of the curve is very similar, but the area under it is 61% higher than that of sTRAIL (10831 ± 132.62).

Specifically, despite having a lower penetration rate, LUV-TRAIL induced death in a higher cell number relative to the total cell number (65% higher for the whole microwell), which confirms LUV-TRAIL it is a more active form of TRAIL.

Interestingly, the lower part of the 3D tumour cell culture where oxygen depletion was more pronounced also correlated with a lower drug efficiency. This difference could be explained by either a lower penetration rate of LUV-TRAIL in this area or a lower efficiency of the drug in a low-oxygen environment. Higher resistance to TRAIL in hypoxic microenvironments has indeed been previously described^47^.

Overall, we have demonstrated that the straightforward and user-friendly configuration of this microdevice allows reproduction of key features of tumours, which should be accounted for in drug testing assays

## Discussion

Here, we reported an SU-8 multi-well microfluidic platform to study the interactions between tumour cells grown in 3D in a collagen matrix in microwells and a tumour-associated endothelium grown on top of the collagen matrix, and for drug testing assays. A key advantage of our design is that it provides easy and precise fluidic control and instantaneous self-filling of the arrays of microwells with viscous hydrogel solutions in one single pipetting step, thanks to the specific geometry of the device with the presence of capillary valves and proper surface hydrophilicity. Therefore, our device fulfils the requirements of being easy to operate by non-specialised users and accessible to the biomedical community, while still allowing recreating complex and physiologically relevant microenvironments. So far, our device comprises 3 independent arrays of microwells, but its design and mode of operation lend themselves well to parallelization for medium to high-throughput assays, which makes our platform particularly interesting for drug testing. Furthermore, the platform is fabricated from SU-8, which presents a low permeability to oxygen, which is advantageous for creating hypoxic environments, as found for instance in tumour tissues. The use of SU-8 for now serves the purpose of developing and optimising the device, however it can later be developed in thermoplastic materials by injection moulding, hence contributing to wide-spreading the technology. Finally, the device is prepared using 90-µm thick substrates, so that it is fully compatible with *in situ* confocal microscopy examination of the cells in the 3D environment, as well as on the top of the microwells.

Here, we successively developed and thoroughly characterised 3D tumour as well as 2D endothelium models, before their combination within one single device. The 3D tumour model faithfully reproduced key features found in tumour tissues *in vivo* such as preferential proliferation for cells with access to more oxygen and nutrients, and creation of an oxygen gradient and a hypoxic core with no cellular proliferation, providing a dense tissue was initially formed. Both healthy and functional endothelium, as well as a leaky endothelium, were successfully prepared in our device, and their integrity and permeability evaluated using immunofluorescence and confocal microscopy. As such, our device can easily mimic vascular integrity in different physiopathological conditions. Interestingly, our study particularly revealed that co-culture of a 3D tumour model with HUVEC cells grown in 2D resulted in the creation of a leaky endothelium, as found in tumour tissues. Next, the established co-culture was used for drug penetration assay across the endothelium and in the tumour tissue and testing of an apoptosis-inducing drug, TRAIL, evaluated here as a soluble formulation (sTRAIL) and a liposome-conjugated formulation (LUV-TRAIL). Our work confirmed that LUV-TRAIL is more efficient in killing cancer cells than its soluble counterpart, despite its lower diffusion rate^48^. In the future, it would be interesting to test other drugs, such as compounds activated in a hypoxic environment, and to include in the 3D cellular models stromal cells and immune cells, which are known to influence the behaviour of tumour cells and to modulate their response to drug treatments.

Altogether, the 3D tumor-2D endothelium co-culture model proposed in this work entails a great potential not only for drug screening and testing of precision medicine, given that only 5-μl samples are required for each assay, but also for more fundamental *in vitro* studies on cellular interactions in complex tumour models. As such, our work is entirely in line with the development and use of enhanced and clinically relevant *in vitro* models, which would allow diminishing the number of experiments conducted on animals and accelerate drug development and approval, hence improving patients’ prognosis and life quality at a faster pace.

## Methods

### Microdevice fabrication and setup

The design of the device includes three independent arrays composed of five different wells each, which are connected in an array by a microchannel (Fig. 1b). As the design was conceived to use capillarity as a driving force to spontaneously fill the whole microdevice, special care was taken to avoid sudden transitions in the microchannel dimensions. Additionally, capillary valves were integrated to constrain the liquid within the microdevice and prevent it from flowing outward the microwells.

The fabrication was carried out as follows: A thin Kapton film (125 µm thickness) was temporarily bonded on top of a Pyrex substrate (Fig. 1b.1). Kapton was used because of its low adhesion to SU-8, allowing easy release of the devices from the substrate after their fabrication. Once the Kapton film was fixed to the substrate, a first SU-8-50 layer was spin-coated on it, followed by a soft-bake treatment, which consists of heating the wafer up to 65 °C for 30 min and cooling it down to room temperature. Next, another SU-8 layer was spin-coated followed by a second soft-bake step. Next, a 140 mJ/cm^2^ exposure dose was applied to cure the first layer of the device using a 365-nm wavelength UV lamp, followed by a post-bake step (Fig. 1b.2). Post-bake step consisted of heating the wafer up to 65 °C for 15 min and cooling it down to room temperature. Following this, the same procedure was repeated to prepare another 80-µm thick layer of SU-8 on top of the first one (spin-coating of 60 µm and 20 µm layers, followed by corresponding soft-bakes). A second step exposure of 140 mJ/cm^2^ through a mask allowed defining the microchannels and microwells (Fig. 1b.3). Then, a post-bake was performed followed by a development step to remove the unexposed SU-8 material. Specifically, the wafer was immersed into an SU-8 developer for 5 min, followed by a rinse step in isopropanol, distilled water and a drying step using nitrogen (Fig. 1b.4). As a result, an open microchannel with microwell structures was fabricated. To close the microchannel and define the microwells, another wafer was processed as before: a 90-µm thick SU-8 layer was processed (spun and soft-baked) on top of another Kapton film temporary bonded to a Pyrex wafer (Figs 1b.2 and 1b.6). Inlets, outlets, and microwells were defined by photolithography using the same exposure and baking parameters as before. The second wafer was finally developed (Fig. 1b.7). Next, both wafers (bottom and cover) were aligned and bonded to each other by applying a pressure of 1 bar and heating up to 90 °C for 15 min (Fig. 1b.8). Finally, the bonded SU-8 devices were manually released from the Kapton (Fig. 1b.9). The final thickness of the device was sufficient to ensure mechanical rigidity and easy handling in the absence of a supporting substrate.

For easy introduction of the hydrogel and hydrogel confinement, the SU-8 was rendered hydrophilic through an oxygen plasma treatment analogous to that described in Jokinen *et al*.^49^, consisting of an 80 s treatment at 300 W, using a pressure of 0.4 mbar and 50% O_2_ to flood the chamber.

### Air-hydrogel interface analysis and optimization in the microdevice

100 μl of hydrogel mixture was prepared as follows to yield a final collagen concentration of 1.2 mg/ml:

36.60 µl of a 3.37 mg/ml stock solution of Type I rat tail Collagen (Corning, 354236) was neutralized by adding 0.9 µl NaOH at 1 M (Sigma 221465). Ionic strength was adjusted by adding 20 µl of DMEM 5x (Sigma, D5030). Finally, the volume was adjusted to 100 μl by adding sterile distilled water. Using a chilled tip, 5 μl droplets of the resulting collagen preparation were placed on top of each inlet to allow spontaneous filling of the microdevice. Afterwards, the microfluidic device was placed in an incubator (37 °C and 5% CO_2_) upside down for 12 min for collagen polymerization.

To assess the effect of evaporation at the hydrogel interface, a 1.2 mg/ml collagen gel was supplemented with 0.2 µm FluoSpheres® (Thermo-Fisher, ref. F-8811) diluted to a final concentration of 1:100 from the stock solution. The resulting collagen mixture was sonicated for 5 min at 4 °C before use to ensure proper dispersion of the Fluospheres®.

### Microdevice biocompatibility and characterization of the 3D cell culture microenvironment

#### Cell isolation and culture

MDA-MB-231 cells, derived from breast adenocarcinoma, were kindly provided by Dr Joan Massagué (Memorial Sloan-Kettering Cancer Centre). MDA-MB-231 cells were routinely grown in high glucose-DMEM culture medium (Lonza BE12-614F) supplemented with 10% FBS (Sigma F7524) and 2 mM L-glutamine (Lonza17-605C) and 1% penicillin (1000 U/ml), 1% streptomycin (1000 µg/ml) (Penicillin/Streptomycin, Lonza DE 17-602E) within a humidified TEB-1000 incubator set at 5% CO_2_, 20% O_2_ and 37 °C (EBERS Medical Technology). Media was refreshed every other day and cells were passaged every 4 days using a Trypsin/EDTA solution (Lonza, CC-5012).

Human Umbilical Vein Endothelial Cells (HUVECs) were extracted at the Miguel Servet Hospital (Zaragoza, Spain) from healthy donors after informed consent according to current ethical regulations. Isolation was performed according to Crampton *et al*.^50^ from umbilical cords 1-3 h after delivery. A fraction of the isolated cells was tested using a FACSAria (BD) flow cytometer to confirm cell positiveness for the endothelial markers CD31, CD105, CD146 and negative for CD45. After testing, HUVEC cells were cultured in EGM-2 culture medium (Lonza CC-3176) prepared according to the manufacturer’s instructions. Only passages 2-4 cells were used for experiments.

The angiogenic potential of HUVEC cells was confirmed as previously reported by DeCicco-Skinner^51^ by seeding the extracted HUVECs on top of a Matrigel® (Corning, 354248) -filled microdevice at a final density of 20,000 cells/cm^2^ (Supplementary Fig. S1).

### Cell culture in microdevices

For 3D cell culture, a 100 μl of hydrogel mixture with a final collagen concentration of 1.2 mg/ml was prepared as before; however, in the last step, the volume was first adjusted to 50 μl by adding sterile distilled water before adding 50 µl of an MDA-MB-231 cell suspension. The resulting cell suspension in hydrogel was carefully homogenized by pipetting up and down several times. Next, the device was filled with the mixture and the hydrogel was polymerized as described above.

To prepare a HUVEC monolayer on top of the collagen in the microwells, HUVEC cells were trypsinized, counted and resuspended in EGM-2. Cells were seeded on top of the microdevice at a final density of 250,000 cells/cm^2^ (saturating concentration). Media was refreshed every other day in the microdevice.

### Cell viability staining

Microdevices were incubated for 15 min in Calcein AM (Invitrogen ref. C3100MP) and propidium iodide (PI) (Sigma-Aldrich, P4170) in Phosphate Buffered Saline (PBS) (Lonza 17-516 F) at final concentrations of 1 μg/ml and 4 μg/ml, respectively, to assess cell viability. Confocal Z-stacks were acquired for each microwell and throughout the entire height of the microwell at 100x magnification.

### Immunofluorescence staining

HUVEC cells in the microdevices were washed with PBS and fixed with 3.7% paraformaldehyde overnight at 4 °C. The following steps were performed at room temperature unless specified otherwise. Cells were permeabilized using 0.1% Triton X-100 (Sigma, T8787) for 5 min and blocked with 1% BSA (Sigma, A1933) for 30 min. Samples were washed twice with 0.05% Tween-20 (Sigma P2287) in PBS between every step.

To quantify endothelium integrity (i.e. the percentage of the microwell surface area covered by HUVEC cells seeded on top of the microdevices), HUVEC cells were stained for VE-cadherin, Actin, and Nuclei. Microdevices were incubated with VE-Cadherin D87F2 XP Rabbit mAb (Cell Signaling 2500 S) in 1% BSA, 0.1% Triton X-100 PBS overnight at 4 °C and washed thrice with 0.05% Tween-20 PBS (Sigma-Aldrich ref. P2287). Next, microdevices were incubated with Alexa Fluor 488 Donkey anti-rabbit IgGs (ThermoFischer A-21206), Phalloidin-TRITC for actin staining and Nucblue® Fixed Cell Stain ReadyProbes® for nuclei staining (Life Technologies R37606) for an hour. Finally, microdevices were washed thrice with 0.05% Tween-20 PBS.

Endothelial integrity in the presence of tumour cells was fluorescently tracked overtime using fluorescence microscopy after staining the HUVEC cells with CMFDA Cell Tracker (Thermo-Fisher C2925). CMFDA was first dissolved at 1 mM in DMSO and used according to the manufacturer’s instructions.

### Oxygen-sensing staining

The variations in the oxygen concentration in the 3D culture within the microdevice was assessed using the oxygen-sensitive dye Image-iT® Hypoxia Reagent (Life Technologies, H10498) and confocal microscopy. This reagent exhibits increased fluorescence upon decrease of the oxygen tension inside the cell. Image-iT® was initially dissolved in DMSO at a concentration of 1 mM and used according to the manufacturer’s instructions.

The fluorescent response of Image-iT® to different oxygen tensions was first calibrated via sodium sulphite addition, which is a known oxygen absorption agent^52^. Cells were plated at 1000 cells/cm^2^ and Image-iT® was added 24 h later. A 2% solution of sodium sulphite (Sigma Aldrich, 08981) was added to yield final concentrations in sodium suphite of 0.25, 0.5, 0.75 and 1%. The fluorescent response of the oxygen-sensitive dye was imaged using confocal microscopy (Supplementary Fig. S3a–c). Its response was further validated with cells pre-conditioned at 1% oxygen overnight in a hypoxia incubator (TEB-100®, EBERS Medical Technologies) (Supplementary Fig. S3d,f).

Cells were embedded in 1.2 mg/ml collagen hydrogel at three different densities (0.4 × 10^6^, 2 × 10^6^ and 10^7^ cells/ml) taking advantage of the independent microwell arrays included in the design. 24 hours after seeding, the oxygen-sensitive dye was added, and cell-induced oxygen gradients within the microwells were imaged for concentrations of 0.4 × 10^6^ and 10^7^ cells/ml.

### Proliferation staining

Cell proliferation was characterised in live cells using the Premo Fucci® Cell Cycle Sensor (Thermo P36237), similarly as in our previous work^53^. Briefly, cells were seeded in 3D as previously described, at three different cell densities (0.4 × 10^6^, 2 × 10^6^ and 10^7^ cell/ml) in the three independent microwell arrays included in the design. Cells were transfected *in situ* 4 h after seeding, using *ca*. 50 virus particles per cell during 24 h. This cell cycle sensor was transduced into the cells by using two different reporters coupled to TagRFP (red) or emGFP (green), which are expressed alternatively during the G1 phase or the S/G2/M phases, respectively.

### Live cell membrane staining

With the aim of distinguishing HUVEC cells from MDA-MB-231 cells when combined in the same microdevice, Vybrant® DiI and DiO were, respectively, used according to the manufacturer’s instructions. Briefly, cells were trypsinized from the culture dish and resuspended in relevant media to inactivate Trypsin. Cells were then pelleted and resuspended in serum-free medium to a concentration of 1 million cells/ml. A solution containing 5 µl of Vybrant® stock per ml of media was added to each cell suspension and cells were incubated with the dye for 15 min at 37 °C in the dark. Finally, cells were thoroughly washed with PBS and resuspended in EGM-2 medium for further use.

### Preparation of soluble recombinant TRAIL and anchoring to lipid vesicles

Once the co-culture model was established, it was applied to test the two TRAIL-based formulations: human soluble recombinant TRAIL (sTRAIL) and Large Unilamellar Vesicles (LUV) with sTRAIL anchored to their surface (LUV-TRAIL). LUV-type liposomes coated with sTRAIL were prepared as previously described^36,54,55^. The lipid composition consisted of a mixture of phosphatidylcholine, sphingomyelin, cholesterol, and 1,2-dioleoyl-sn-glycero-3-[[N-(5-amino-1-carboxypentyl)-iminodiacetic acid]succinyl](nickel salt) (Avanti Polar Lipids) in a 55:30:10:5 weight ratio to mimic the human exosome lipid composition. sTRAIL corresponding to amino acids 95–281 cloned into the pET-28c plasmid (Novagen - kindly provided by Dr. M. MacFarlane)^19^ with a 6-histidine tag was attached to the liposome surface as previously described^56^. Generated LUV-TRAIL was characterised as described elsewhere^54^ and stored at 4 °C until use.

### Drug diffusion assays

To assess the penetration rate across the endothelium and through the hydrogel for the different compounds, the microdevices were prepared as described in previous sections and filled with collagen as described in sections 2.1 and 2.2. Microdevices were covered with a 4 mg/ml TRITC-LUVs, 30 μM TRITC 70 kDa Dextran solution or 30 μM FITC 40 kDa Dextran solution, and Z-stack confocal images were taken for at least 2 h throughout the entire microwell height. The diffusion rate was calculated as the change in position of the 50% intensity value over the observed time.

### Imaging

Phase contrast and fluorescence microscopy images were taken using a Nikon Eclipse Ti® inverted fluorescence microscope equipped with a C1 modular confocal microscope system. To acquire whole microwell images, 250-μm thick confocal stacks were performed, by taking images every 10 μm. Finally, a thermostated chamber within the microscope was equilibrated to 37 °C and 5% CO_2_ whenever live cell imaging was performed.

### Image analysis and processing

Image analysis was performed using Fiji® (http://fiji.sc/Fiji). In order to analyse cell viability, viable and dead cells were counted manually, whereas cell distribution and marker expression were quantified as the percentage of the microwell area where the marker was being expressed. Finally, cell circularity was assessed manually for at least 20 cells in each sample.

### Statistical analysis

Results are presented as the mean ± standard error. The normal distribution was tested by the Kolmogorov-Smirnov test. Statistical significance was set at p < 0.05. For nonparametric comparisons, a Kruskal-Wallis test was performed followed by the Mann-Whitney U-test.

## Electronic supplementary material

Supporting information
