# Supplementary material for: Multiwell capillarity-based microfluidic device for the study of 3D tumour tissue-2D endothelium interactions and drug screening in co-culture models
Source: Sci Rep. 2017 Sep 20;7:11998. doi: 10.1038/s41598-017-12049-4 (PMC5607255; doi:10.1038/s41598-017-12049-4)
Supplement: Supplementary file 1 — Supporting information [file 41598_2017_12049_MOESM1_ESM.pdf]

Multiwell capillarity-based microfluidic device for the study of 3D tumour tissue-2D endothelium interactions and drug screening in co-culture models.

María Virumbrales-Muñoz[a,b,c], José María Ayuso[e,f], Marta Olave[a,b,c], Rosa Monge[a,b,c,d], Diego de Miguel[g,h,i], Luis Martínez-Lostao[i,j,k,l], Séverine Le Gac[m], Manuel Doblare[a,b,c], Ignacio Ochoa[a,b,c,*], Luis J. Fernandez[a,b,c,*]

a. Group of Applied Mechanics and Bioengineering (AMB). Centro de Investigación Biomédica en Red. Bioingenieria, biomateriales y nanomedicina (CIBER-BBN), Spain.

b. Aragon Institute of Engineering Research (I3A), University of Zaragoza, Spain.

c. Aragon Institute of Biomedical Research, Instituto de Salud Carlos III, Spain.

d. BEONCHIP S.L., Zaragoza, Spain.

e. Medical Engineering, Morgridge Institute for Research, 330 N Orchard street, Madison, WI, USA. 53715.

f. Department of Biomedical Engineering, Wisconsin Institutes for Medical Research, University of Wisconsin-Madison, 1111 Highland Avenue, Madison, Wisconsin 53705, United States.

g. Centre for Cell Death, Cancer and Inflammation (CCCI), UCL Cancer Institute, University College of London, UK.

h. Department of Biochemistry, Molecular and Cell Biology, University of Zaragoza, Spain.

i. Aragon Institute of Biomedical Research (IIS Aragón), Instituto de Salud Carlos III, Spain.

j. Department of Microbiology, Preventive Medicine and Public Health, University of Zaragoza, Spain.

k. Department of Immunology, University Clinical Hospital Lozano Blesa, Zaragoza, Spain.

l. Institute of Nanoscience of Aragón (INA), Zaragoza, Spain.

m. Applied Microfluidics for BioEngineering Research, MESA+ Institute for Nanotechnology, MIRA Institute for Biomedical Research and Technical Medicine, University of Twente, The Netherlands.

*These authors equally coordinate this project.

Corresponding authors: Luis Fernandez-luisf@unizar.es. Ignacio Ochoa- iochgar@unizar.es

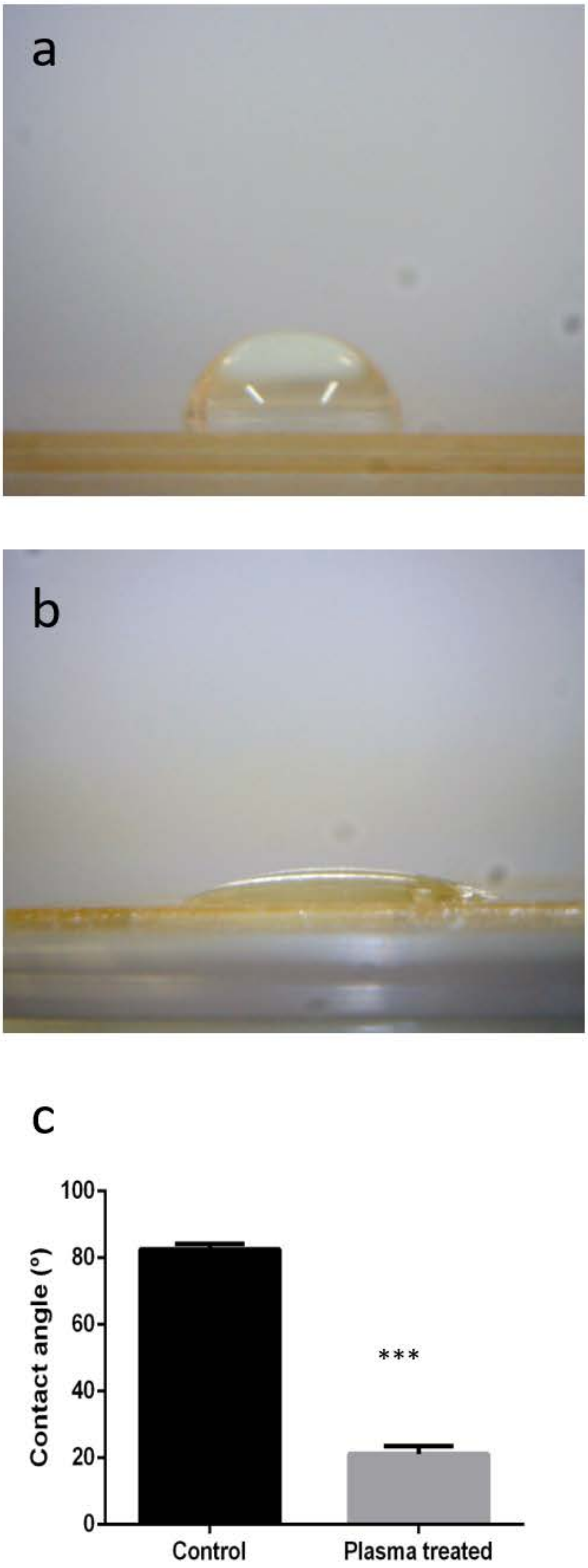
a
b
c
Contact angle (°)
100
80
60
40
20
0
***
Control
Plasma treated

Supporting Figure S1. Contact angle measurements for the SU-8 material without (a) and after (b) O_2 plasma treatment. Measurements were performed at least 6 independent times for each condition. Data distribution was found not normal and (***) P-value was 0.0007 as calculated through Kruskal Wallis' Test.

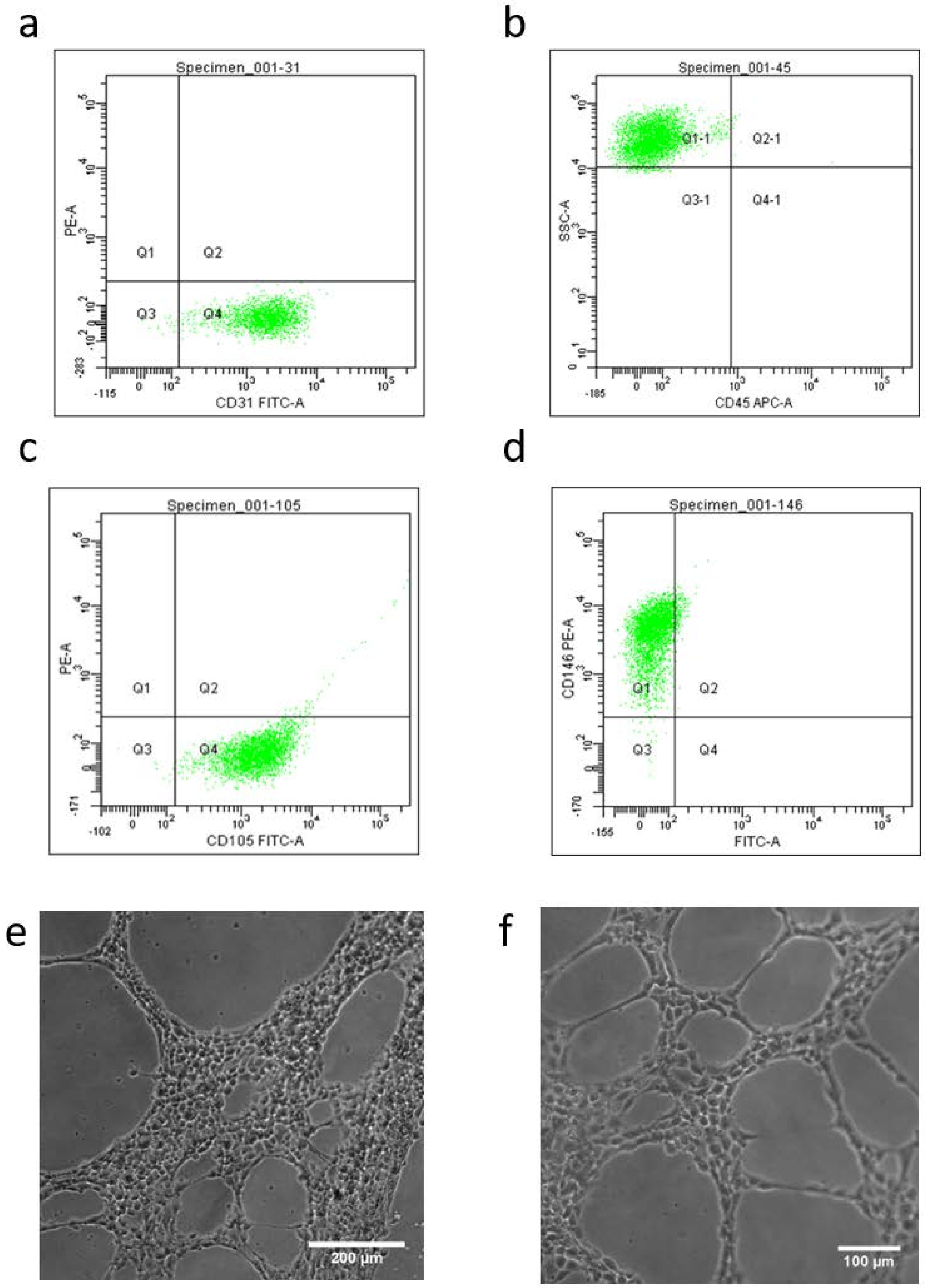

Supporting Figure S2. Phenotypical characterization of the extracted HUVEC cells to confirm their endothelial lineage. Flow cytometry was performed for the markers CD31 (a), CD45 (b), CD105 (c) and CD156 (d). Results are consistent with HUVEC cells. Furthermore, cells were cultured in Matrigel to check their angiogenic potential through the tube formation assay (e and f). Results confirm the extracted cells are HUVECs.

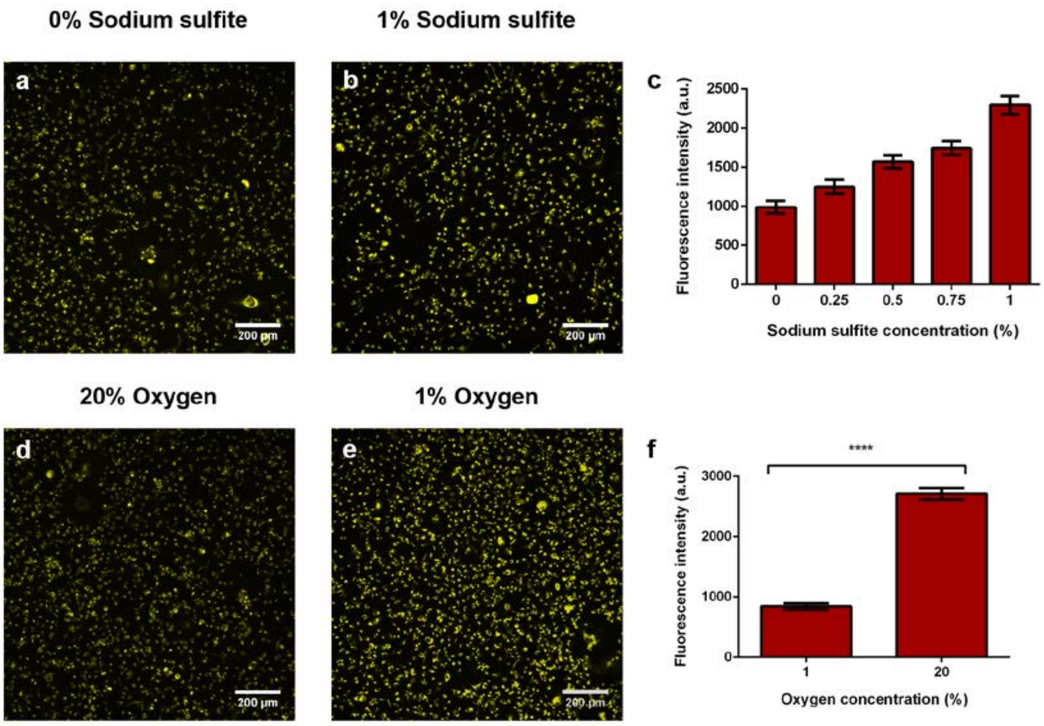

Supporting Figure S3: Validation of the fluorescent response of the oxygen-sensitive reagent Image-iT®. Cells were treated with the reagent and cultured under different concentrations of sodium sulfite (0, 0.25, 0.5, 0.75 and 1%). Results for 0% (a) and 1% (b) of sodium sulfite are shown. Fluorescence was quantified an plotted for the described concentrations (c). The reagent signal was also compared for cells incubated under atmospheric conditions (d) and 1% oxygen € overnight. Data were normal (f), and found significantly different (****) p-value < 0.0001 as calculated through Student's t-test (n=3).

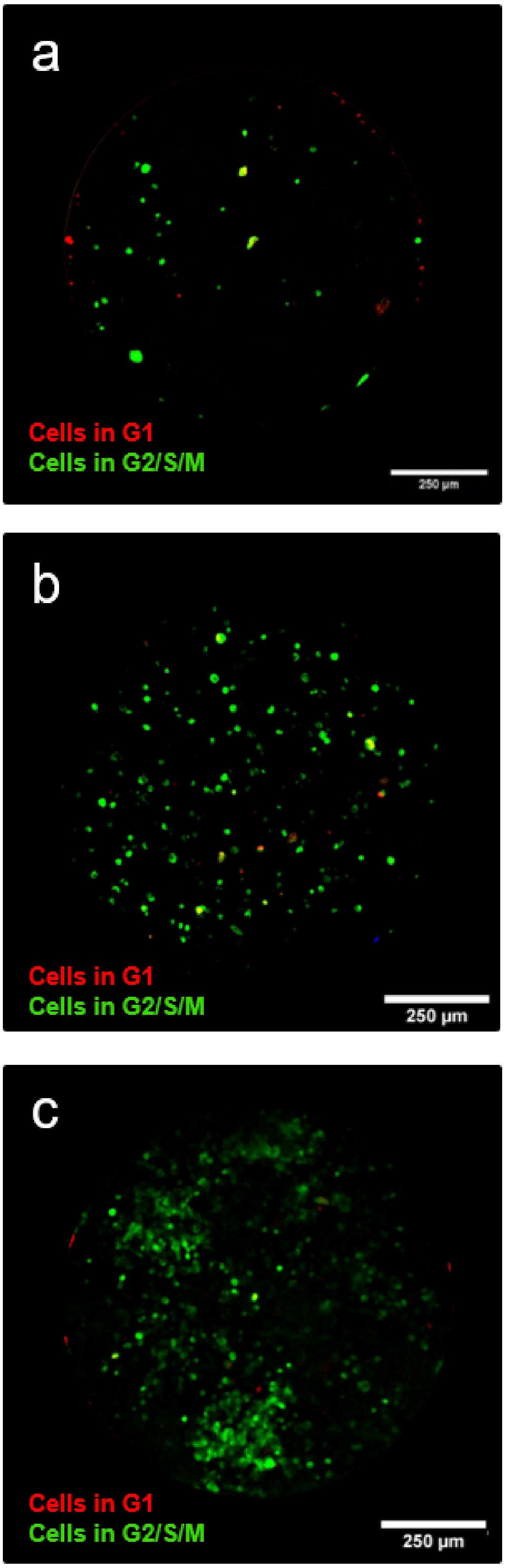

Supporting Figure S4. Whole microwell images of cell proliferation. Assessment was carried out through Premo FUCCI® transfection for different cell densities 0.4·10^6 (a), 2·10^6 (b) and 10^7 cells/ml (c). Green cells are in the G2/S/M phases, whereas red cells are arrested in the G1 phase (non-proliferating cells).

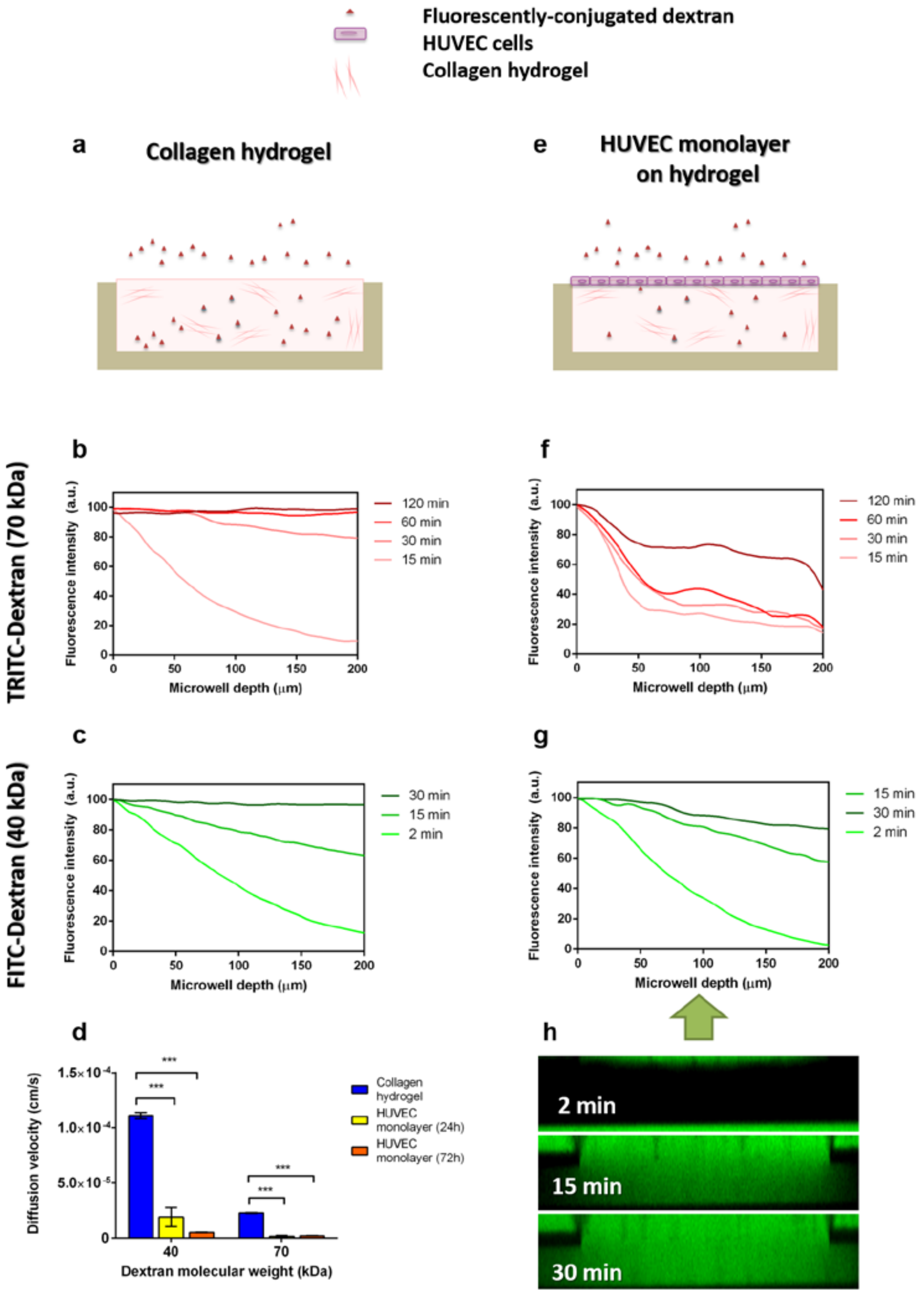

Supporting Figure S5: Characterization of endothelial permeability. Schematic representation of the diffusion experiments through a control hydrogel (a) and an endothelial monolayer on a collagen hydrogel (e). Diffusion profiles for a TRITC 70 kDa dextran through a control hydrogel (b) and an endothelial monolayer after 24 hours of seeding (f). Diffusion profiles for a FITC 40 kDa dextran through a control hydrogel (c) and an endothelial monolayer after 24 hours (g). Quantification of diffusion rates for the described dextrans through a control hydrogel and an endothelial monolayer after 24 and 48 hours of seeding. Data were not normally distributed and significant differences, as found through Kruskal-Wallis' test, (***) were found (p-value < 0.0001) (n=3). Sample of a diffusion profile for TRITC 40 kDa dextran through an endothelial monolayer (g).

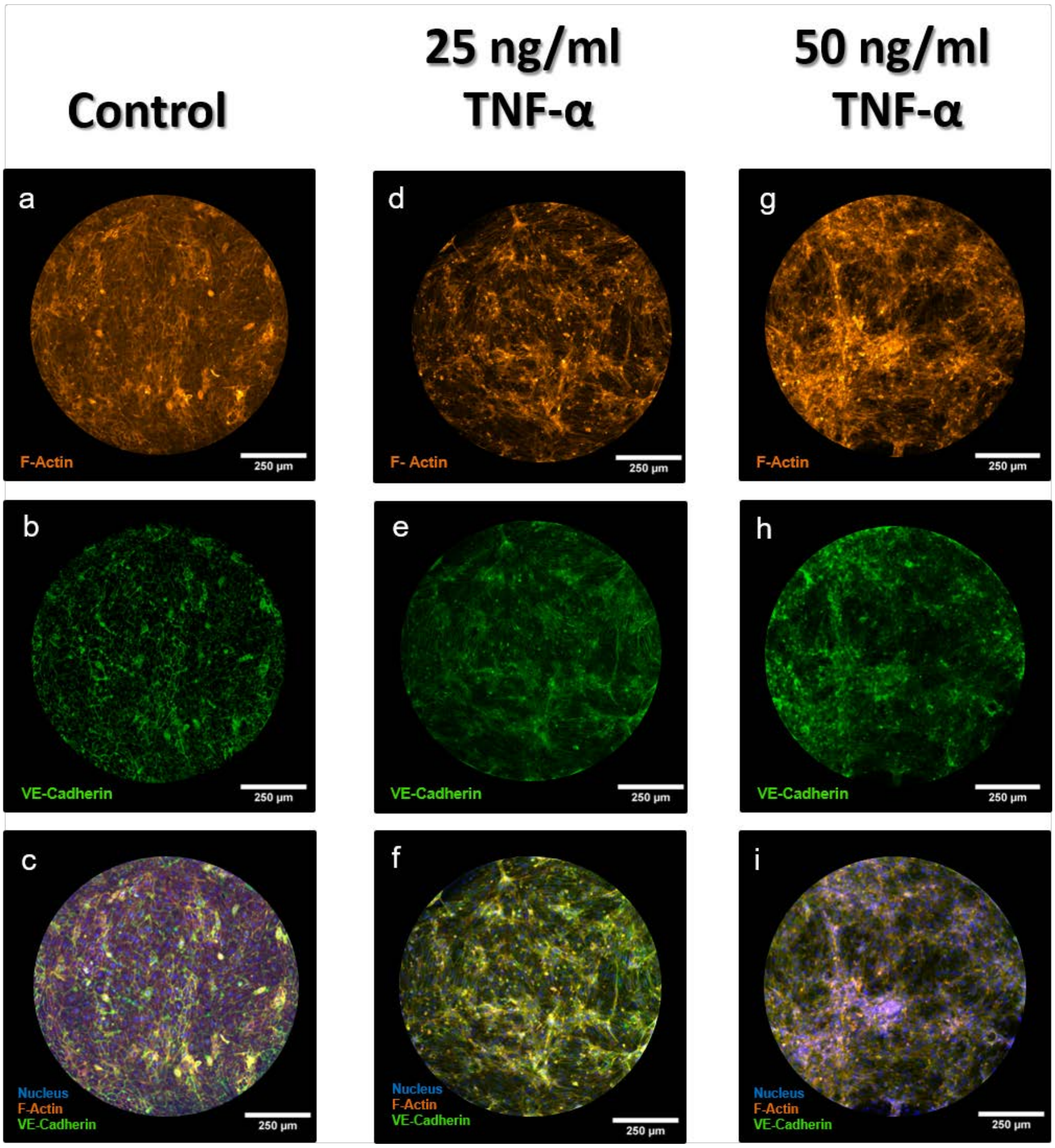

Supporting Figure S6. Whole microwell immunofluorescence images to verify the endothelium integrity. Images show F-actin, VE-cadherin and Merge (F-actin, VE-cadherin and DAPI) for Control conditions (a, b and c, respectively); cells supplemented with 25 ng/ml of TNF-α (d, e and f, respectively) and cells supplemented with 50 ng/ml of TNF-α (g, h and i, respectively).

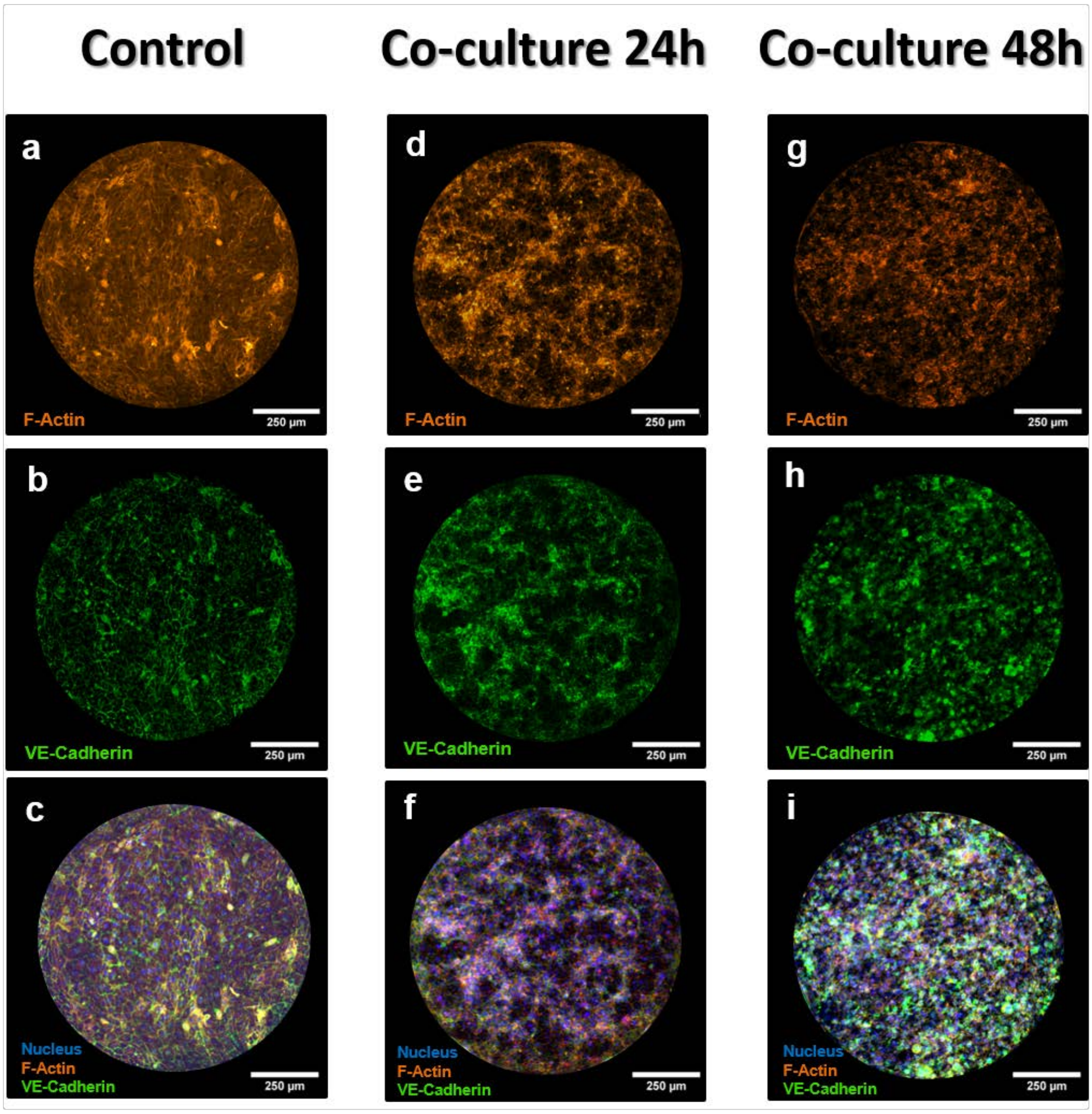

Supporting Figure S7. Cancer-endothelium interaction effects on the endothelium. Whole microwell immunofluorescence images to assess endothelium integrity. Images show F-actin, VE-cadherin and Merge (F-actin, VE-cadherin and DAPI) for Control conditions (a, b and c, respectively); cells in co-culture with MDA-MB-231 cells for 24 hours (d, e and f, respectively) and cells in co-culture for 48 hours (g, h and i, respectively).

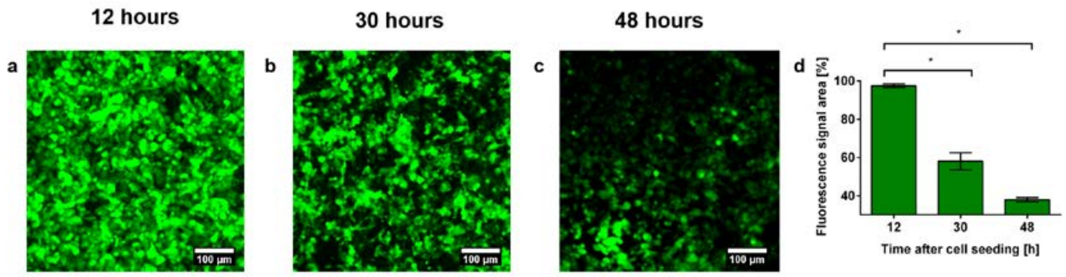

Supporting Figure S8: Tracking of the integrity of the endothelium in co-culture. Cells were tracked using CMFDA to assess endothelium integrity at 12 (a), 30 (b) and 48 hours (c) after seeding. Area was quantified (d) and found to significantly decrease overtime. Data were not normal and significant differences were found (*) $p<0.05$ through Kruskal-Wallis' test (n = 3).

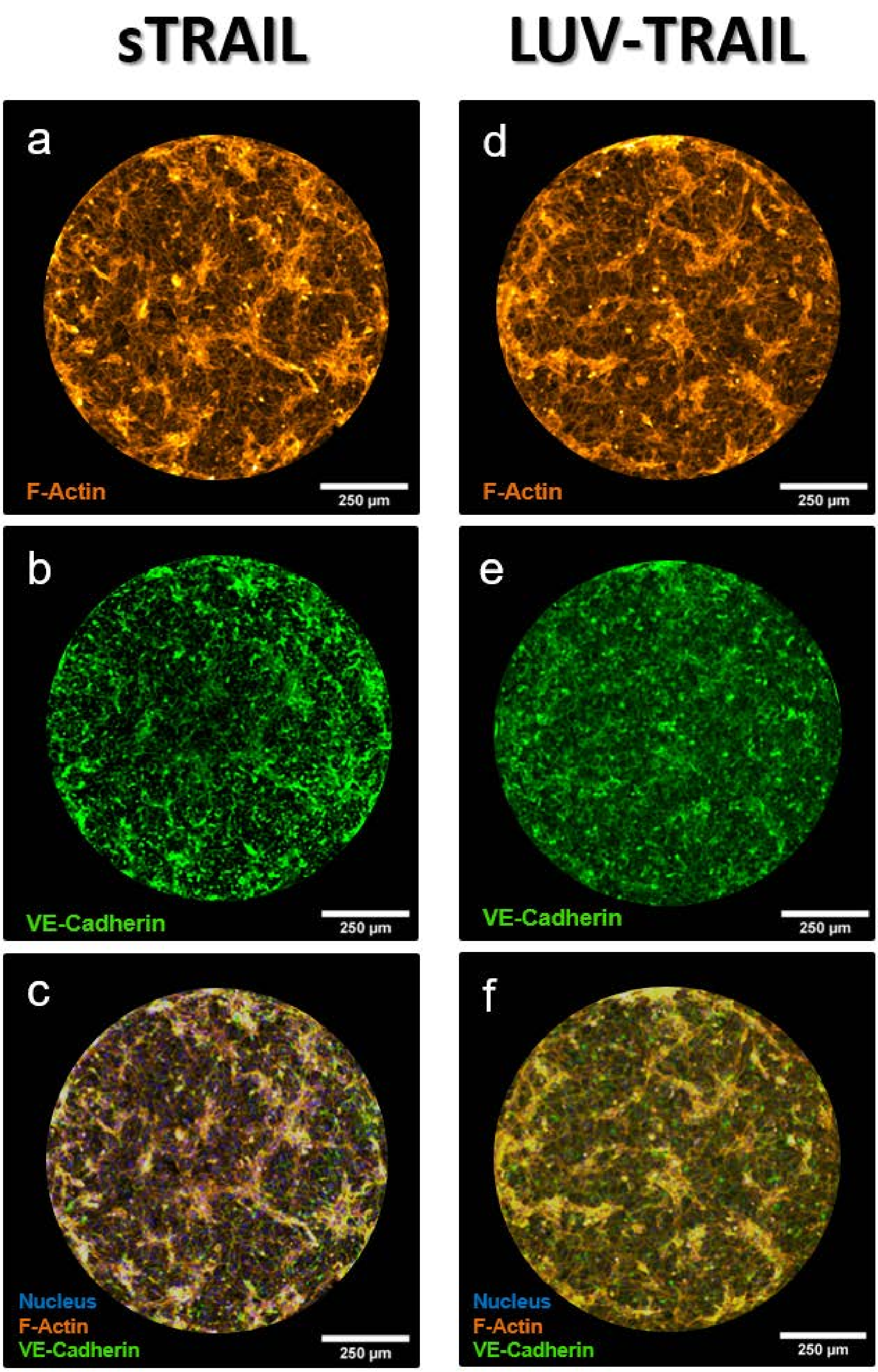

Supporting Figure S9. Whole microwell immunofluorescence images to verify the endothelium integrity after exposure to the TRAIL drug. Images show F-actin, VE-cadherin and Merge (F-actin, VE-cadherin and DAPI) for sTRAIL (a, b and c, respectively) and LUV-TRAIL (d, e and f, respectively). Images were taken after 24 hours of treatment. Concentration for both drugs was 0.33 ng/ml.
